# Low COVID-19 vaccine uptake in people living with HIV and those with hypertension and diabetes without HIV at Mbarara and Masaka regional referral hospitals: A cross-sectional survey

**DOI:** 10.1371/journal.pgph.0003270

**Published:** 2024-05-23

**Authors:** Asiphas Owaraganise, Brian Beesiga, Jaffer Okiring, Michelle E. Roh, Elijah Kakande, Joan Nangendo, Cecilia Akatukwasa, Jordan John Lee, Florence Mwangwa, Jane Kabami, Fred C. Semitala, Moses R. Kamya

**Affiliations:** 1 Infectious Diseases Research Collaboration, Kampala, Uganda; 2 Department of Internal Medicine, Makerere College of Health Sciences, Kampala, Uganda; 3 Department of Epidemiology and Biostatistics, University of California, San Francisco, San Francisco, California, United States America; 4 Division of Infectious Diseases and Geographic Medicine, Department of Medicine, Stanford University, Santa Clara, California, United States of America; 5 Department of Epidemiology and Population Health, Stanford University, Santa Clara, California, United States of America; 6 Makerere University Joint AIDS Program, Kampala, Uganda; Universite de Montreal, CANADA

## Abstract

Chronic diseases such as HIV, hypertension, and diabetes increase the risk of severe coronavirus disease 2019 (COVID-19) and death. Thus, COVID-19 vaccine uptake data among these priority populations are needed to inform immunization programs. We assessed COVID-19 vaccine uptake among people living with HIV (PLWH) and those with hypertension/diabetes without HIV (PWoH) in Southwestern and Southcentral Uganda and determined factors influencing vaccination. We conducted a cross-sectional study from January to April 2023. We enrolled a random sample of participants aged 18 years and older seeking HIV, hypertension, or diabetes care at two regional referral hospitals (RRHs) in Mbarara and Masaka in Uganda. Using vaccination records abstraction and interviewer-administered questionnaires, we collected data on COVID-19 vaccine uptake, sociodemographic data, and reasons for non-uptake in unvaccinated persons. We compared COVID-19 vaccination uptake between PLWH and PWoH and applied modified Poisson regression to determine sociodemographic factors associated with vaccine uptake. The reasons for non-vaccine uptake were presented as percentages. Of the 1,376 enrolled participants, 65.6% were fully vaccinated against COVID-19. Vaccination coverage was 65% among PWLH versus 67% among PWoH. Higher education attainment and older age were associated with COVID vaccination. Participants with secondary education and those aged ≥50 years achieved >70% coverage. Fear of side effects was the most cited reason (67%) for non-vaccination among 330 unvaccinated participants, followed by vaccine mistrust (24.5%). People with chronic diseases in Southwestern Uganda had slightly lower than 70% COVID-19 vaccine coverage as recommended by WHO. Higher educational attainment and older age were linked to increased vaccine uptake. However, mistrust and fear of vaccine side effects were the main reasons for non-vaccination. To increase COVID-19 vaccine uptake, programs must reach those with lower educational attainment and younger age groups, and address the fear of vaccine side effects and mistrust among persons with underlying diseases in Uganda.

## Introduction

Coronavirus disease 2019 (COVID-19) is a respiratory disease caused by severe acute respiratory syndrome coronavirus 2 (SARS-CoV-2) that emerged in late 2019 and remains a significant threat to global health security [1]. The COVID-19 pandemic caused approximately 3 million excess deaths [2] and disrupted global mobility and trade [3]. People with underlying chronic diseases, such as persons living with HIV (PLWH) and those with diabetes, cancer, heart disease, and hypertension, are at an increased risk of persistent SARS-CoV-2 infections [4–6] and severe COVID-19 comorbidities and death [7–10]. In Africa, COVID-19 cases rose slowly at first but surged significantly in mid-2020 with the emergence of new variants, especially Delta and Omicron, which mainly affected people with underlying health conditions [11–13]. Also, COVID-19-related deaths doubled in countries with a higher burden of underlying chronic diseases [14]. Likewise, human immunodeficiency virus (HIV) and other immunocompromising chronic diseases could foster the spread of SARS-CoV-2 variants of concern [4, 8] due to attenuated vaccine response [15]. Non-immune individuals could become SARS-CoV-2 reservoirs, leading to future COVID-19 outbreaks with emerging variants.

Significant progress was made in containing and spreading COVID-19 using novel COVID-19 vaccines in the last quarter of 2020. To control the COVID-19 pandemic, the World Health Organization (WHO) recommended coverage of COVID-19 vaccines to at least 70% of the population in all countries around the globe by mid-2022 [16]. During the vaccination rollout, priority was given to vulnerable groups, such as those with underlying diseases such as HIV, hypertension, diabetes, and heart disease [17–19]. However, as of July 4, 2023, the African continent with the largest PLWH population [20, 21] had administered the fewest COVID-19 vaccines—approximately 41 doses per 100 individuals versus the global average of 154 per 100 individuals [22]. By July 07, 2023, Uganda had administered about 57 COVID-19 vaccine doses per 100 individuals [23].

Effective strategies for controlling and mitigating the intersecting effects of duo HIV/COVID-19 pandemics require data-driven approaches tailored to specific contexts that accelerate the delivery of protective and lifesaving COVID-19 vaccines [24–26]. Studies have shown that COVID-19 vaccination uptake has been impacted by demographic, institutional factors, and geographic location both within and between countries around the globe [27–29]. While the initial Ugandan policy limited COVID-19 vaccine eligibility to adults with chronic diseases and workers in healthcare, education, and security sectors, updated data on COVID-19 vaccination among PLWH and persons with hypertension or diabetes without HIV (PWoH) are currently limited. Additionally, it is unclear how COVID-19 risk perception, vaccine availability, stigma and fear of vaccine side effects, and mistrust in vaccine impacted vaccine uptake despite being cited as barriers to COVID-19 vaccination [19, 30, 31] in the general population. This study aimed to determine i)COVID-19 vaccine uptake among persons living with HIV and those with hypertension or diabetes without HIV attending health clinics in Southwestern and Southcentral Uganda, ii)factors associated with COVID-19 vaccine uptake, and iii) reasons for non-uptake.

## Methods

### Study design and duration

We used a cross-sectional design to compare the proportions of COVID-19 vaccine uptake and its determinants between PLWH and PWoH receiving medical care for diabetes and hypertension at Masaka and Mbarara regional referral hospitals (RRHs) from January 10^th^ to April 24^th^ 2023.

### Study setting

Masaka and Mbarara RRHs are located in Southwestern and Southcentral Uganda, regions where HIV prevalence remains high (average 7.9% of the population) [32]. The hospitals serve approximately 472,629 people in Mbarara and 297,004 in Masaka [33]. They host the largest HIV clinics in each respective region and belong to the International Epidemiological Databases to Evaluate AIDS (IeDEA)—East Africa region—a multicenter cohort study that brings together data from 53 HIV care and treatment sites in 3 countries to examine critical clinical and operational issues surrounding the scale-up of services in East Africa [34]. The AIDS Support Organization (TASO), funded by The U.S. President’s Emergency Plan for AIDS Relief (PEPFAR), and the AIDS Healthcare Foundation-funded Uganda Cares support the Masaka HIV clinic. In contrast, the PEPFAR supports the Mbarara HIV clinic. Both HIV clinics utilise an open Electronic Medical Records System (EMRS), making their data complete and reliable for data abstraction. Each hospital runs specialised out-patient clinics for chronic diseases such as hypertension and diabetes. The approximate HIV clinic volume is 15,200 and 12,300 for Masaka and Mbarara hospitals, respectively. Approximately 2,500 and 3,200 people are enrolled in hypertension and diabetes clinics at Masaka and Mbarara RRH, respectively, funded by the government.

### Study population and eligibility criteria

We enrolled adults with hypertension (HTN) or diabetes (DM) without HIV and adults receiving care for HIV at Mbarara and Masaka Hospitals after March 10, 2021, when COVID-19 vaccination started in Uganda [23]—approximately one year from vaccination rollout to study start. Men and women aged 18 years and over were eligible for the study, excluding those who did not provide informed consent.

### Sample size estimation, sampling procedure, and data collection

We estimated 1,600 subjects, 800 per group, to detect a 7% difference in proportions of COVID-19 vaccination in adult PLWH and PWoH receiving chronic care using a population vaccination prevalence of 0.498 [35], power of 0.8, type I error probability alpha of 0.05, sample size allocation ratio of 1:1 and design effect of 2 for sampling from two hospitals.

Due to time constraints, we could only recruit 1,376 participants. We then re-evaluated the power analysis and determined that achieved sample size was sufficient to assess associations between vaccination uptake and related factors. We therefore analyzed 1,376 participants, which was adequately powered to detect a 7% difference in proportion, accounting for double the number of people living with HIV compared to those without HIV at the study sites using the observed vaccination prevalence of 66.8%. We queried the electronic medical record (EMR) database categorised by HIV status at each hospital and generated a random list of individuals using the clinic IDs of those with HIV and without HIV. We then used the random list to systematically enrol those who met the study inclusion criteria per category of PLWH and PWoH. When a participant was deemed ineligible on review due to declining consent or having chronic diseases other than HIV, hypertension, and diabetes, the next eligible individual from the list was selected until the required sample size was achieved.

After obtaining informed consent, we collected data on sociodemographic characteristics, medical history, and COVID-19 vaccination status using a structured questionnaire. For non-vaccinated participants, we asked them to select the reason(s) for not getting vaccinated from a list of four options: vaccine stockout, low self-perceived COVID-19 risk, distrust in vaccine effectiveness, or fear of vaccine side effects.

### Study outcome and variables

The primary outcome was the proportion of complete vaccination uptake, defined by self-report and verified from the national COVID-19 vaccination card or vaccination register. These standard registers captured comprehensive COVID-19 vaccine data, including the manufacturer’s brand (e.g., Oxford-AstraZeneca, Sinovac-CoronaVac, Pfizer-BioNTech, Moderna and Johnson & Johnson’s Janssen), date of each dose, telephone number, national identity number, and biodata. The secondary outcomes were reason for non-vaccination defined as the percentage of self-reported reason among those who had not received any COVID-19 vaccine. Sociodemographic characteristics such as age in completed years, sex (female or male), occupation (essential workers, farmers, commerce and trade, and unemployed), education level (primary, secondary and post-secondary/tertiary meaning completion of high school diploma including attending university), marital status (yes for married or no for divorced/separated/widowed), study sites of Mbarara and Masaka RRHs, and HIV status were evaluated as potential factors associated with COVID-19 vaccine uptake.

### Data management and statistical analysis

Trained research assistants collected and entered data into Research Electronic Data Capture (REDCap) database. Co-investigators and the study biostatistician performed routine data checks. Analyses were conducted using STATA version 14.0 (StataCorp, College Station, Texas, U.S.A.)

Descriptive characteristics were summarized using proportions categorized as PWoH and PLWH. Socio demographic factors known to be associated with vaccine uptake from the literature [36] were included in a multivariable model. Modified Poisson regression with robust standard errors [37] was used to assess factors associated with COVID-19 vaccination. Results were presented as prevalence ratios (PR) with corresponding 95% confidence intervals (CI). We followed the Strengthening the Reporting of Observational Studies in Epidemiology (STROBE) reporting guideline (S1 Checklist).

### Ethical considerations

The study was approved by the Makerere University School of Medicine Research and Ethics Committee (SOMREC), approval number Mak-SOMREC-2021-201, and the Uganda National Council for Science and Technology (UNCST), approval number HS1855ES. The study was conducted per the Declaration of Helsinki principles. We obtained a waiver of consent to screen patient records to determine eligibility and study sampling. Written informed consent was obtained before participant enrollment.

## Results

### Participant sociodemographic characteristics

Of the 1,376 enrolled participants, 59.5% (n = 819) were PLWH (Table 1). A nearly equal proportion of participants were sampled from Masaka and Mbarara RRHs. The majority of participants were female (73.8%; n = 1,015), and median age was 47 years (interquartile range (IQR): 38, 55). PWoH were older than PLWH, with 62% of PWoH participants ≥50 years of age compared to 19.9% of PLWH).

**Table 1:** Descriptive characteristics of participants with chronic underlying diseases stratified by HIV status at Masaka and Mbarara RRHs, Jan-April 2023.

Characteristic	Category	HIV status	
		Negative (n = 557) n (%)	Positive(n = 819) n (%)
Hospital (cluster)	Masaka	290 (52.1)	388 (47.4)
	Mbarara	267 (47.9)	431 (52.6)
Age bracket	30 or below	11 (2.0)	114 (13.9)
	31–40 years	50 (9.0)	282 (34.4)
	41–50 years	149 (26.8)	260 (31.7)
	50 or above	347 (62.3)	163 (19.9)
Sex	Male	134 (24.1)	227 (27.7)
	Female	423 (75.9)	592 (72.3)
Occupation	Essential workers	48 (8.6)	148 (18.1)
	Farmer	363 (65.2)	395 (48.2)
	Commerce and trade	107 (19.2)	230 (28.1)
	Unemployed	39 (7.0)	46 (5.6)
Education level	None/Primary	390 (70.0)	586 (71.6)
	Secondary	122 (21.9)	190 (23.2)
	Tertiary/University	45 (8.1)	43 (5.2)
Married	No	18 (3.2)	64 (7.8)
	Yes	539 (96.8)	755 (92.2)

### COVID-19 vaccine uptake

**Fig 1:**
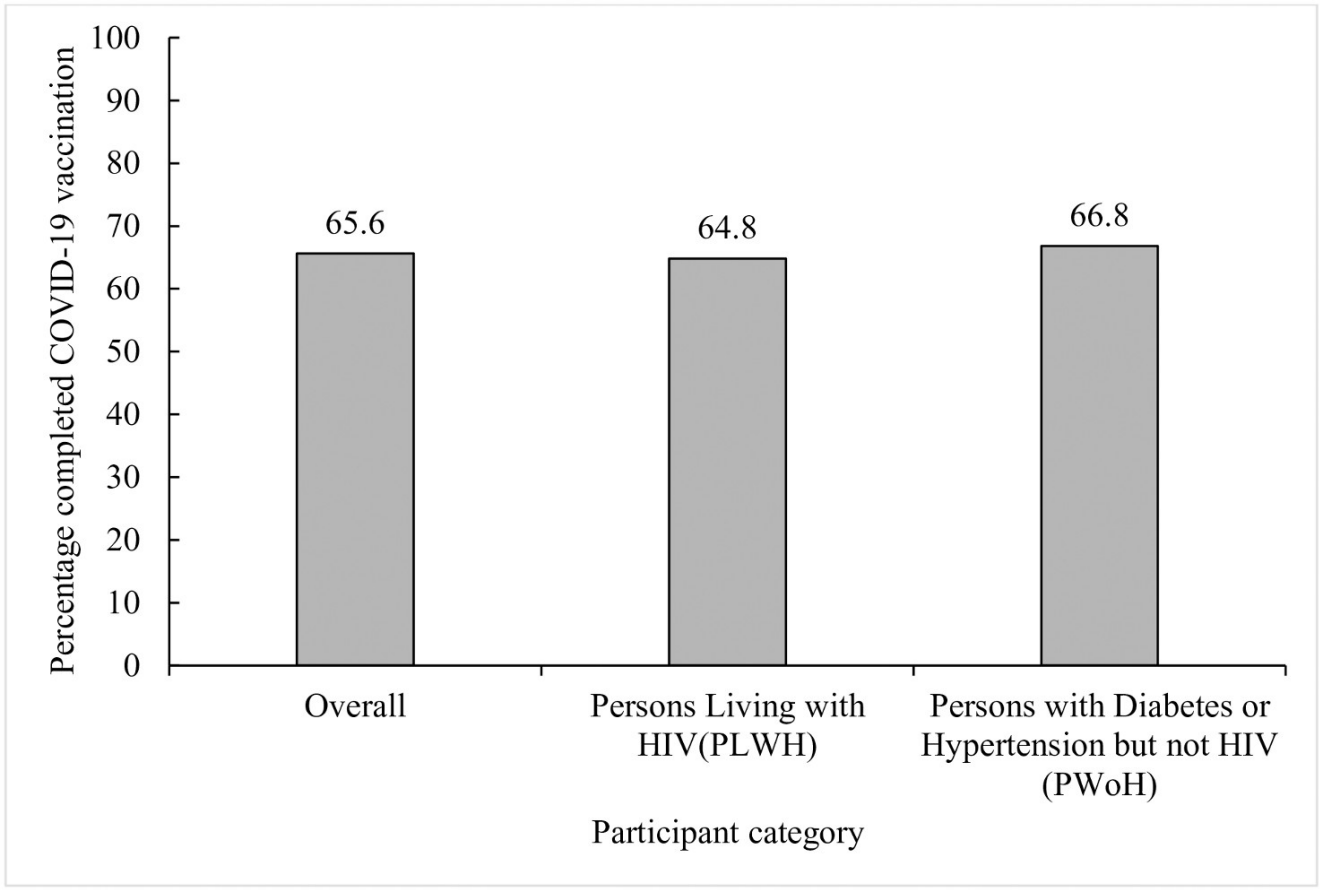
COVID-19 vaccine uptake among participants with underlying chronic diseases stratified by HIV status.

Overall, 65.6% [95% CI: 63.1, 68.1] of participants completed their COVID-19 vaccination series (Fig 1). When stratified by HIV status, 64.8% [95% CI: 61.5, 68.0] of PLWH were fully vaccinated compared to 66.8% [95% CI: 62.8, 70.6] of PWoH; these differences were minimal.

### Factors associated with COVID-19 vaccine uptake

Based on our multivariable analysis, factors positively associated with COVID-19 vaccine uptake were being older than 50 years compared to 30 years and below (PR = 1.34, 95% CI: 1.12,1.61) and seeking care at Mbarara as compared to Masaka RRH (PR = 1.15, 95% CI: 1.06, 1.39) (Table 2). Also, tertiary (PR = 1.24 [95% CI: 1.08, 1.43]) or secondary (PR = 1.21, 95% CI: 1.11, 1.32) education levels as compared to primary or no education was associated with COVID-19 vaccine uptake. These subgroups reached >70% vaccine coverage.

**Table 2:** Factors associated with COVID-19 vaccine uptake in participants with underlying chronic diseases at Mbarara and Masaka RRHs, Jan-April 2023, N = 1,376.

Characteristic	Category	Vaccinated	Multivariable analysis	
		n (%)	Crude PR (95% CI)	Adjusted PR (95%CI)
Hospital/Cluster	Masaka	406 (59.9)	Reference	Reference
	Mbarara	497 (71.2)	1.19 (1.10, 1.29)	1.15 (1.06, 1.25)
Age	≤30	70 (56.0)	Reference	Reference
	31–40	206 (62.1)	1.11 (0.93, 1.32)	1.12 (0.94,1.33)
	41–50	261 (63.8)	1.14 (0.96, 1.35)	1.14 (0.96,1.37)
	≥51	366 (71.8)	1.28 (1.09, 1.51)	1.34 (1.12,1.61)
Education	None/Primary	604 (61.9)	Reference	Reference
	Secondary	230 (73.7)	1.19 (1.10, 1.29)	1.21 (1.11, 1.32)
	Tertiary/University	69 (78.4)	1.27 (1.12, 1.43)	1.24 (1.08, 1.43)
Sex	Male	241 (66.8)	Reference	Reference
	Female	662 (65.2)	0.98 (0.90, 1.06)	1.06 (0.97, 1.16)
Occupation	Essential workers	136 (69.4)	Reference	Reference
	Farmer	502 (66.2)	0.95 (0.86, 1.06)	0.96 (0.85, 1.09)
	Trade	209 (62.0)	0.89 (0.79, 1.01)	0.91 (0.80, 1.04)
	Unemployed	56 (65.9)	0.95 (0.79, 1.14)	0.98 (0.82, 1.18)
Married	No	51 (62.2)	Reference	Reference
	Yes	852 (65.8)	1.06 (0.89, 1.26)	1.07 (0.90, 1.28)
HIV status	PWoH	372 (66.8)	Reference	Reference
	PLWH	531 (64.8)	0.97 (0.90, 1.05)	1.07 (0.98, 1.16)

PR = Prevalence Ratio

### Reason for non-uptake of COVID-19 vaccines

**Fig 2:**
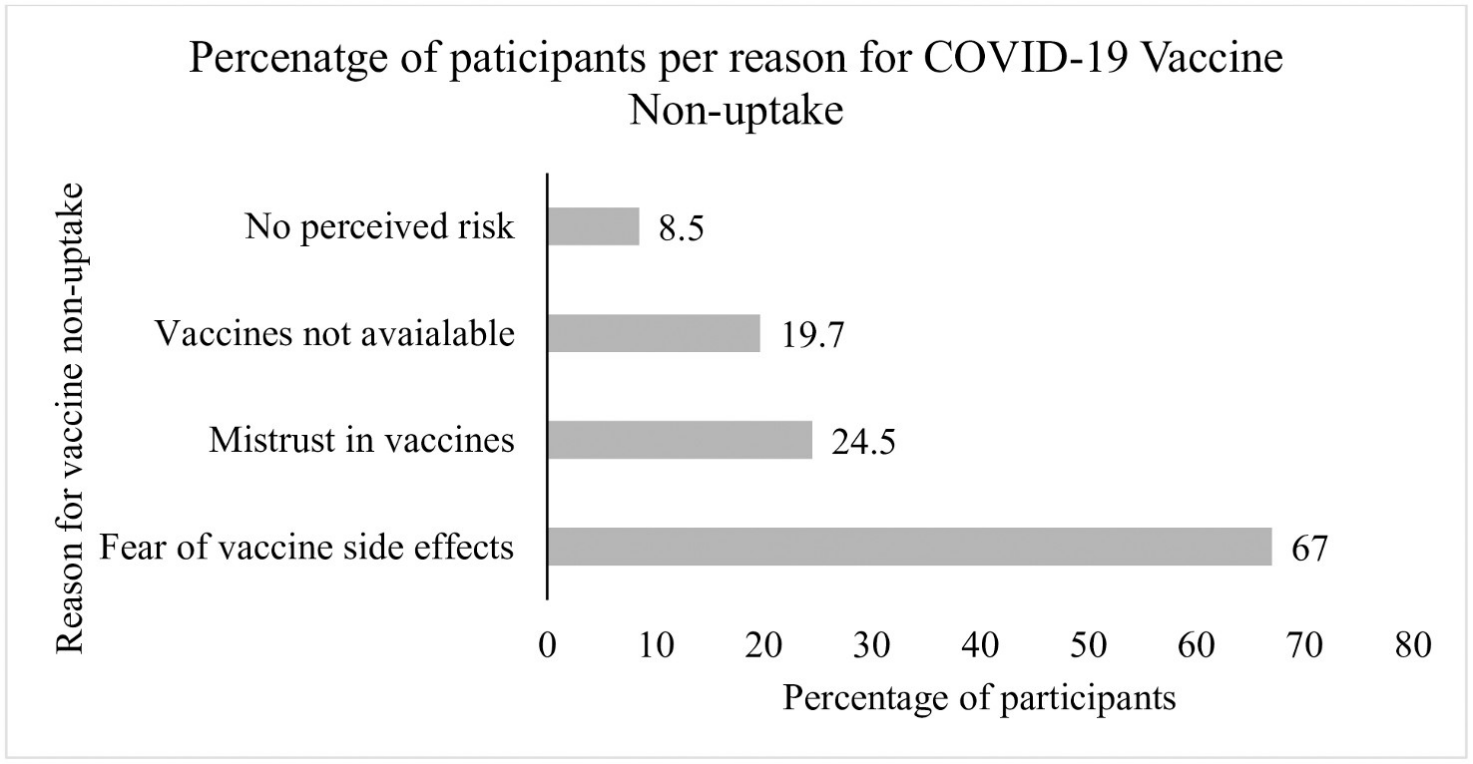
Reasons for non-vaccination in participants with underlying chronic diseases.

Among non-vaccinated participants (n = 330), fear of vaccine side effects was the highest (67%) cited reason for non-uptake of the COVID-19 vaccine, followed by mistrust in vaccines (24.5%), vaccines not available 19.7% and low risk perception 8.5% (Fig 2).

## Discussion

### COVID-19 vaccination uptake among persons with underlying chronic diseases

In this cross-sectional study, 65.6% of people living with underlying chronic diseases in Southwestern and Southcentral Uganda completed COVID-19 vaccination, failing to reach the WHO recommendation of ≥70% population coverage [16]. However, >70% vaccine uptake was seen among those with secondary (73.7%) and tertiary (78.4%) education and those ≥50 years of age (71.8%), a known high-risk group for severe COVID-19 [38]. It is important to note that 65.6% of COVID-19 vaccine uptake illustrates an improvement from the 50% coverage reported in the 2020 national online survey [39]. Among those who were unvaccinated, the primary reasons were fear of the vaccine’s side effects and mistrust.

Our findings are consistent with a 2022 study from Uganda that reported a 69% uptake of at least one dose of the COVID-19 vaccine among PLWH [31], but inconsistent with a 2022 study in Ethiopia [40] reported 29.6% COVID-19 vaccine uptake among persons with chronic diseases. The possible explanations for the suboptimal vaccine uptake include challenges in vaccine distribution, the potential influence of misinformation, and the overall trend of declining COVID-19 pandemic impact [41]. Other reasons may include lifting previous COVID-19 restrictions, including full operation of public transport, decreased use of social distancing and masks, and increased complacency towards protective behaviour, including vaccine uptake. In addition, the conflicting messaging on the severity of COVID-19 among PLWH could have lowered individual risk perception [42] and, subsequently, the uptake of COVID-19 vaccines. The suboptimal COVID-19 vaccination uptake emphasizes a need for intensified efforts from national governments and partners in resource-limited settings with prevalent underlying chronic diseases to achieve the WHO COVID-19 vaccination targets.

### Factors associated with COVID-19 vaccination in persons with underlying chronic diseases

Our findings showed that higher educational attainment was positively associated with higher COVID-19 vaccine uptake, confirming several other studies conducted in central Uganda [28], Nigeria [43], and Kenya [44] that showed an overall positive association between COVID-19 vaccine uptake and higher educational attainment. Higher educational attainment could have made it easier for participants to learn more about vaccine risks and benefits while at the same time discerning between disinformation and misinformation that characterised the COVID-19 response measures. It is also possible that employers who mandated vaccination contributed to the higher vaccination rates among individuals with higher education [45]. Higher COVID-19 vaccination in participants aged above 50 years is also consistent with the literature [44]. Finally, we found that participants attending Mbarara RRH were more likely to be fully vaccinated than those attending Masaka RRH. This regional variation in COVID-19 vaccination could have been partly due to the differential rollout of information and actual COVID-19 vaccine rationing that characterised the inception of the COVID-19 vaccination programs. Immunization campaigns may need to target younger individuals and those with lower levels of education who may underestimate their risk for severe COVID-19.

### Reasons for COVID-19 vaccine non-uptake in persons with underlying chronic diseases

We found that among non-vaccinated participants, fear of vaccine side effects ranked highest. This result is consistent with reasons for the non-uptake of booster COVID-19 vaccine dose, including uncertainty about vaccine safety and efficacy and unclear information on eligibility and availability [46]. However, this finding differs from complacency or vaccine inaccessibility reported by WHO as the top global threats to immunisation programs [47]. Differing COVID-19 vaccine mandates and skepticism from political and social media may have partly led to mistrust in vaccine side effects among non-vaccinated persons. Clinicians need to educate persons with underlying chronic diseases on emerging vaccine data and recommendations, including clarifying and countering misinformation and disinformation that further deter vaccine-hesitant individuals.

Our study had some strengths and limitations. We prospectively interviewed a large random sample of PLWH persons with underlying chronic conditions who face the highest risk of severe COVID-19 outcomes due to intersecting COVID-19 and HIV pandemics, which improves reliability of our findings. However, our study had a few limitations. First, the study was conducted during a brief period (between January and April 2023) and may have missed any trends occurring with COVID-19 vaccine uptake. Furthermore, despite cross-checking self-reported data with vaccination registers and cards, misclassification and social desirability biases were possible. Finally, while the study was conducted at the largest hospitals in Southwestern and Southcentral Uganda, our findings may not be generalizable to regions outside of the southern Uganda and at other health facilities without the robust resources for access to care.

In summary, despite the importance of COVID-19 vaccination among persons with underlying chronic diseases, this cross-sectional study found that overall, 66% were fully vaccinated. Older age groups (i.e., ≥50 years of age) and those with secondary education and above had the highest vaccination uptake, reaching the WHO goal of ≥70% vaccine coverage. Being more educated or in care at Mbarara RRH was also positively associated with COVID-19 vaccine uptake. Fear of side effects and mistrust in the vaccine were the primary reasons for non-vaccination. Thus, immunization programs aimed at improving COVID-19 vaccination among people with underlying chronic diseases should target the less-educated and younger age groups with sensitization campaigns that address the fear of vaccine side effects and misinformation about vaccines.

## Supporting information

S1 Checklist STROBE statement—Checklist of items that should be included in reports of *cross-sectional studies*. (DOCX)

S1 Data (DTA)
